# Salt-Induced Gel Formation by Zwitterionic Polymer for Synergistic Methane Hydrate Inhibition

**DOI:** 10.3390/gels11080637

**Published:** 2025-08-12

**Authors:** Fei Gao, Shijun Tang, Peng Xu, Jiancheng Wu, Xinru Li

**Affiliations:** 1 School of Petroleum Engineering, Yangtze University, National Engineering Research Center for Oil & Gas Drilling and Completion Technology, Wuhan 430100, China; fgat163@163.com (F.G.); lixinru0102@163.com (X.L.); 2 Hubei Key Laboratory of Oil and Gas Drilling and Production Engineering, Yangtze University, Wuhan 430100, China; 3 State Key Laboratory of Low Carbon Catalysis and Carbon Dioxide Utilization, Yangtze University, Wuhan 430100, China; 4 Turpan Oil Production Plant, PetroChina Tuha Oilfeld Company, Hami 839000, China; tangsjtlf@petrochina.com.cn; 5 Daqing Oilfield NO. 9 Production Plant, Daqing 163000, China; dq_wujiancheng@petrochina.com.cn

**Keywords:** zwitterionic polymer, hydrate inhibition, salt-induced physical gel structure, response surface method, deep-water drilling

## Abstract

In deepwater drilling operations, inhibiting methane hydrate (MH) formation is critical for wellbore flow assurance. This study synthesized a zwitterionic polymer NDAD and evaluated its hydrate inhibition performance through high-pressure reactor tests, magnetic resonance imaging (MRI), and molecular simulations. Results demonstrate that NDAD at concentrations of 1.0 wt% extends MH formation time by 4.9 times compared to conventional inhibitor PVCap. Notably, NaCl (10–15 wt%) synergistically enhances inhibition efficiency by inducing NDAD chain stretching to form physical gel networks, increasing solution viscosity by 98%. This gel structure obstructs methane–water diffusion, prolonging hydrate induction time. Response surface methodology (RSM) identifies NDAD dosage as the primary control factor for inhibition efficacy. Molecular simulations confirm that NDAD inhibits hydrate formation through dual pathways: (i) competitive hydration by ionic groups disrupting water cage structures and (ii) gel networks imposing mass transfer resistance to methane diffusion.

## 1. Introduction

With the increasing demand for energy sources due to economic development, it is crucial to explore new sources of fossil fuels [1,2]. Deep-sea sediments are typically abundant in oil and gas resources, offering broad prospects for exploration and development [3,4]. The South China Sea, as one of the largest offshore oil and gas regions in the world [5,6]. However, the complex and harsh geological conditions pose significant technical challenges to drilling fluids in deep-water drilling operations [7,8,9]. During deep-water drilling, methane hydrates are prone to forming in the wellbore due to the high-pressure and low-temperature environmental conditions, posing potential risks of wellbore fluid assurance accidents [10]. Over the past decades, researchers have introduced various chemical for MH formation inhibition [9,11,12].

Traditional hydrate inhibitors include two types: thermodynamic inhibitors and kinetic inhibitors [13,14]. Among them, thermodynamic inhibitors include chemicals such as salts and methanol/ethylene glycol [14,15]. However, thermodynamic inhibitors rely on high doses (20–60 wt%) to suppress hydrate formation [16,17], thus facing environmental pressure and cost issues. Kinetic inhibitors are chemicals that can effectively inhibit the formation of hydrates at low doses [18,19,20]. China National Offshore Oil Corporation has developed N-vinyl caprolactam/vinyl isobutyl ether copolymer. By matching the hydrate cage structure with the hydrophobic ring spacing, the adsorption efficiency has been increased by 30% [19,20,21]. Under a subcooling temperature of 7.5 °C, the induction time was over 200 min, and the gas consumption was reduced by 75%, breaking through the low-temperature failure bottleneck of traditional KHIs [22]. In addition, the combined use of antifreeze proteins and thermodynamic inhibitors, by forming a self-assembling protective layer, increases the inhibition efficiency by 40% compared to pure chemical methods. Traditional hydrate inhibitors achieve adsorption with hydrates by anchoring hydrophobic molecules in empty half-cages on the surface of hydrates [23]. Earlier molecular simulations of the poly (vinyl caprolactam) (PVCap) and growing hydrate surface indicated that PVCap could adsorb onto different planes of the hydrate by connecting multiple hydrophobic rings to adjacent or distant cages [24]. However, the hydrophobic groups of the polymer adsorbed in the cages on the surface of the hydrate are likely to enhance the structure around the cages, thereby causing the adsorption force of the adjacent cages to be stronger than that of the isolated monomer.

Zwitterionic polymers are polymer materials in which both cationic and anionic groups are present in their polymer chains [18,25]. The zwitterionic groups exhibit strong ionic hydration, which can disrupt the cage structure of water molecules in MHs to a certain extent, showing potential for inhibiting hydrate formation [23,26]. Additionally, zwitterionic groups can inhibit clay hydration through ionic adsorption and polymer film-forming properties. Therefore, zwitterionic polymers exhibit dual-functional inhibition performance in inhibiting both hydrate formation and clay hydration, demonstrating great potential in deep-water drilling.

Aiming at the problem of the formation and blockage of natural gas hydrates in the wellbore during deepwater drilling, which leads to drilling accidents, an amphoteric ionic polymer was prepared. The high-pressure reactor and magnetic resonance imaging methods were used to prove that this amphoteric ionic polymer has a very good inhibitory effect on hydrate formation. Further research was conducted on the feasibility of salt-induced formation of a gel structure in this amphoteric polymer, thereby further increasing the mass transfer resistance of the polymer and hindering the formation of hydrates. In addition, the synergistic effects of stirring rate, temperature and subcooling degree were explored by using the response surface method(RSM). Finally, the mechanism by which amphoteric ionic polymers inhibit hydrate formation was explored based on molecular simulation methods.

## 2. Results and Discussion

### 2.1. Optimization of Preparation Conditions

#### 2.1.1. Single Factor Tests

Firstly, the single-factor analysis method was used to optimize the preparation conditions of NDAD, and then the orthogonal experiment method was employed to further optimize the optimal preparation conditions of NDAD with the best hydration inhibition and hydrate inhibition performance.

**Figure 1:**
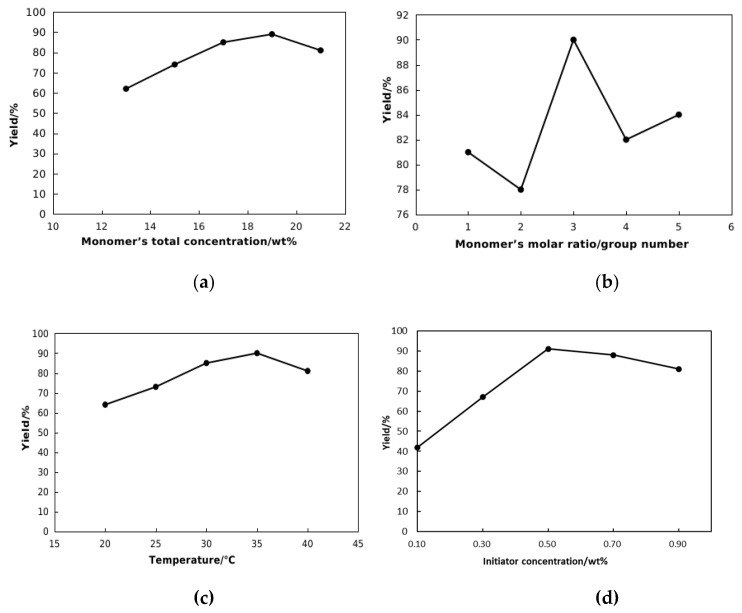
Variation in NDAD yield over (**a**) monomer’s total concentration; (**b**) monomer’s molar ratio(group number); (**c**) temperature; (**d**) initiator concentration.

Total monomer concentration: In order to determine the optimal total concentration of reactive monomer for preparation of NDAD, the molar ratio of fixed reactive monomer in the experiment was NVCL:DMAA:AMPS: DMDAAC(Full spellings can be seen in the table abbreviation) = 3:3:2:2, pH value was 7, reaction temperature was 25 °C, and initiator mass fraction was 0.30%. Polymerization reaction was performed at 13%, 15%, 17%, 19%, and 21%, and then the polymerization yield was tested. The experimental results are shown in Figure 1. As can be seen from Figure 1a, with the gradual increase in the total concentration of reactive monomer, the polymerization yield increases first and then decreases. When the concentration is 19%, the synthesized product has the largest polymerization yield. Therefore, the optimal total monomer concentration for the preparation of NDAD was determined to be 19%.

Monomer’s molar ratio: In order to determine the optimal proportion of reactive monomers for the preparation of NDAD, the total concentration of reactive monomers was fixed at 19%, pH value was 7, reaction temperature was 25 °C and initiator mass fraction was 0.30%. The polymerization reaction was carried out under five different molar ratios of monomers, and then the polymerization yield was tested. Labeled as experimental groups 1 to 5, the molar ratios of reactive monomers NVCL:DMAA:AMPS: DMDAAC are, respectively, 3:3:2:2, 4:4:1:1, 4:2:2:2, 2:4:2:2, and 2:2:3:3. The experimental results are shown in Figure 1b. As can be seen from Figure 1, when the molar ratio of reaction monomer NVCL:DMAA:AMPS: DMDAAC of the synthesized product is 4:2:2:2, the polymer yield is the highest. Therefore, the optimal molar ratio NVCL:DMAA:AMPS: DMDAAC for the preparation of NDAD was determined to be 4:2:2:2.

The influence of the reaction temperature: In order to determine the optimal reaction temperature for preparing NDAD, the molar ratio of the fixed reaction monomer was NVCL:DMAA:AMPS: DMDAAC = 4:2:2:2, pH value was 7, the total concentration of monomer was 19%, and the mass fraction of initiator was 0.30%. The polymerization reaction was carried out at the reaction temperature of 20 °C, 25 °C, 30 °C, 35 °C and 40 °C, and then the polymerization yield was tested. The experimental results are shown in Figure 1c. With the gradual increase in reaction temperature, the polymerization product increases first and then decreases. When the reaction temperature is 30 °C, the synthesized product has the largest polymerization yield. Therefore, the optimal reaction temperature for preparing NDAD was determined to be 35 °C.

The influence of initiator concentration: In order to determine the optimal initiator concentration for preparation of NDAD, the molar ratio of reaction monomer was maintained NVCL:DMAA:AMPS: DMDAAC =4:2:2:2, and pH value was chosen as 7, and the total reaction monomer concentration was 19% and the reaction temperature was 25 °C. The initiator system is prepared with same molar ratio of (NH_4_)_2_S_2_O_8_/NaHSO_3_. The polymerization reaction was carried out at different initiator concentrations of 0.1%, 0.3%, 0.5%, 0.7% and 0.9%, and then the polymerization yield was tested. The experimental results are shown in Figure 1. As can be seen from Figure 1d, with the initiator concentration increasing gradually, the polymer yield increases first and then decreases. When the initiator concentration is 0.5%, the synthesized product has the largest polymerization yield. Therefore, the optimal initiator concentration for preparing NDAD was determined to be 0.5%.

#### 2.1.2. Orthogonal Tests

According to the principle of free radical polymerization and the results of single-factor tests, the main factors affecting NDAD polymerization are the total monomer concentration, molar ratio of monomer used, reaction temperature and initiator concentration. Orthogonal tests with four factors (A~D) and three levels were designed. Table 1 is the factor level table of the orthogonal test, and Table 2 shows the results of orthogonal tests.

According to the design conditions in Table 1, 16 kinds of target products were synthesized, and their yield, hydrate inhibition rate and clay hydration inhibition rate were tested according to the test method of target products. The experimental results are shown in Table 2. When processing the experimental results, the experimental data of yield rate, hydrate inhibition rate and hydrate dissociation rate were first standardized and denoted as X, Y and Z, respectively. Then the three evaluation indexes were assigned the same weight, H being the comprehensive evaluation index, H = X + Y − Z. The larger the H, the higher the polymer yield, and also the better the hydrate inhibition and clay hydration inhibition. From the analysis results in Table 2, it can be seen that the main factors affecting the comprehensive effect of the target product of the polymerization reaction are total concentration of monomer > molar ratio of monomer > reaction temperature > initiator mass fraction. According to the mean analysis results, the optimal synthesis conditions were A3, B4, C3, and D2; that is, the monomer molar ratio NVCL:DMAA:AMPS: DMDAAC = 4:2:2:2, the total monomer concentration was 19%, and the initiator mass fraction was 0.50% (as a percentage of the total mass of the monomer) at a reaction temperature of 35 °C.

### 2.2. Molecular Structure Characterization of NDAD

**Figure 2:**
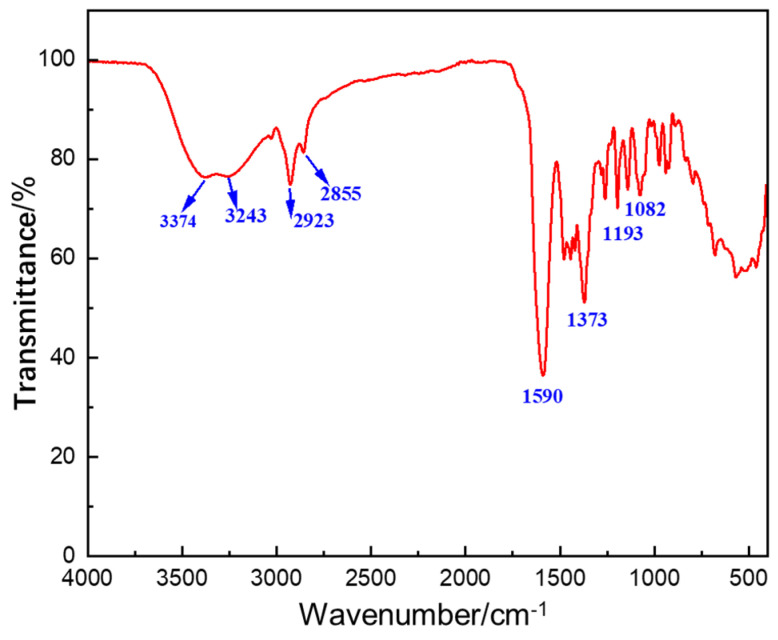
The Fourier-transform infrared (FTIR) spectra of NDAD.

The chemical structure of the copolymer NDAD was initially verified by Fourier transform infrared spectroscopy (FTIR). Characteristic absorption bands corresponding to all four monomer units were clearly observed in the Figure 2. The peak at 3374 cm^−1^ belongs to the N-H stretching vibration of the primary amine group (-NH_2_) in the DMAA molecule. The peak at 3243 cm^−1^ corresponds to the N-H stretching vibration of the primary amine group (-NH_2_) in the AMPS molecule. The absorption peak at 1590 cm^−1^ is a characteristic peak of the caprolactam ring skeleton vibration. The stretching vibration absorption peaks of the sulfonic acid group (-SO_3_H) in the AMPS molecule are located at 1082 cm^−1^ and 1193 cm^−1^, respectively. The peak presented at 1373 cm^−1^ is assigned to the vibration of C–N in DMDAAC, and 2855 cm^−1^ is the absorption peak of the stretching vibration of the five-membered ring, suggesting that DMDAAC opens the double bond connection to form a ring.

**Figure 3:**
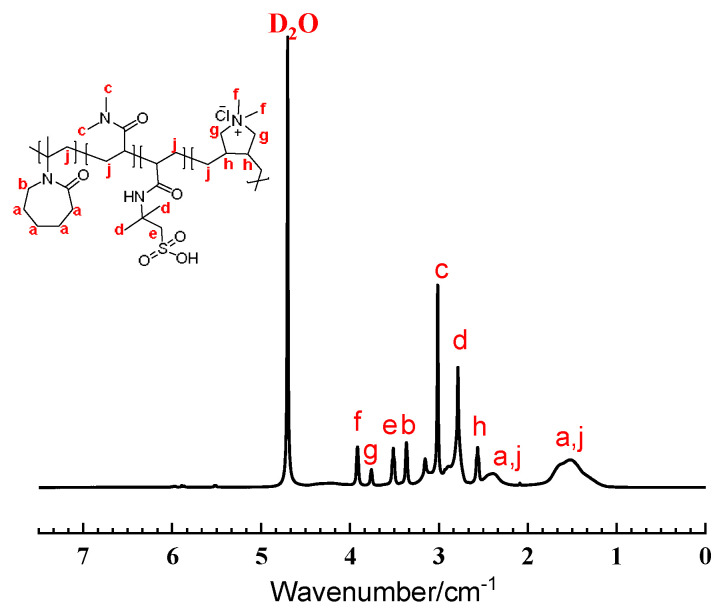
^1^H NMR spectra of NDAD samples in D_2_O at 25 °C.

Further structural confirmation was obtained through ^1^H NMR spectroscopy, as shown in Figure 3. The spectrum exhibited characteristic proton signals from each monomer: The methylene protons adjacent to the amide group in NVCL resonated at 3.37 ppm, while the dimethylamino protons of DMAA appeared as a singlet at 3.01 ppm. The methyl protons of AMPS adjacent to the sulfonic acid group showed resonance at 2.79 ppm, and the allylic protons from DMDAAC were observed at 3.91, 3.74, and 2.55 ppm. The absence of vinyl proton signals (5.0–6.5 ppm) confirmed complete consumption of monomers during copolymerization.

The molecular weights and polydispersities (PDI) of the NDAD were obtained. Gel permeation chromatography (GPC) tests were conducted using standard polystyrene as the standard sample and N, N-dimethylformamide (DMF) as the mobile phase. The weight-average molecular weight (Mw) of NDAD was measured to be 141,843 g/mol. The number-average molecular weight (Mn) is 56,782 g/mol, and its molecular weight distribution index (Mw/Mn) is 2.498.

### 2.3. Hydrate Formation Inhibition Performance of NDAD

**Figure 4:**
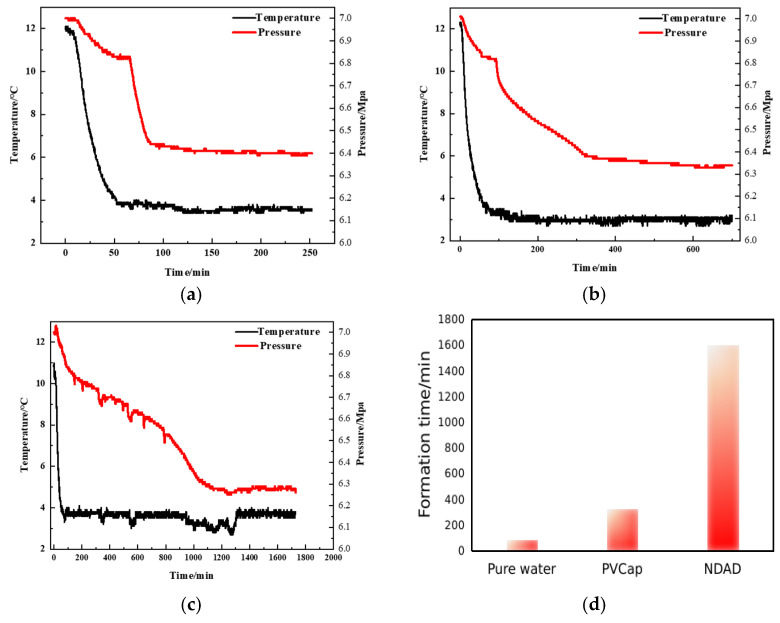
The pressure-time curves of hydrate formation in (**a**) pure water; (**b**) 1%PVCap and (**c**) 1%NDAD; (**d**) Comparison of hydrate formation time of pure water, PVCap and NDAD.

The hydrate formation times of pure water, commercial hydrate inhibitor PVCap (Poly(N-vinylcaprolactam), and NDAD aqueous solutions were evaluated using hydrate formation equipment under a high pressure and low-temperature environment. As shown in the Figure 4, the results show that the formation process of hydrates can generally be divided into three stages. The first stage: the nucleation stage of hydrate crystals. It is characterized by a slow decrease in pressure followed by a tendency to level off in pressure-time curves. This change can be explained by the fact that methane in the high-pressure vessel gradually dissolves in water first and participates in the nucleation process of hydrates, leading to the slow drop of pressure in the vessel. When methane dissolves to a certain extent, the pressure remains basically unchanged. It can be explained by the fact that hydrates are not generated in large quantities during this process but form crystal nuclei in the solution. As the reaction time progressed, the pressure curve decreased rapidly. After that, the pressure continued to decrease slowly until it tended to be stable, meaning that the hydrates were basically completely formed. Observing the Figure 4, the formation process of hydrates basically follows these three stages. For a pure water system, the nucleation process of the hydrate lasted for 65 min. Within 65~86 min, the pressure dropped from 6.8 MPa to 6.4 MPa, indicating that hydrates were forming rapidly. However, the addition of inhibitors can significantly prolong the nucleation or formation time of hydrates. According to the experimental results, the hydrates’ formation time in aqueous solutions with PVCap was significantly prolonged. The nucleation time was extended to 92 min, and the formation process of hydrates was extended to 326 min, which was much longer than the hydrates formation time in pure water. Surprisingly, the hydrate inhibitor NDAD exerts a more excellent performance. From the perspective of the pressure-time curves, the hydrate formation time was significantly extended to 1270 min in aqueous solutions with NDAD. After that, the hydrate gradually formed, and the formation time was also delayed to 1600 min. The hydrate formation time of NDAD is ten times that of pure water, which greatly delays the formation of hydrates. This result is of great significance for wellbore flow insurance in the process of deepwater oil and gas development.

**Figure 5:**
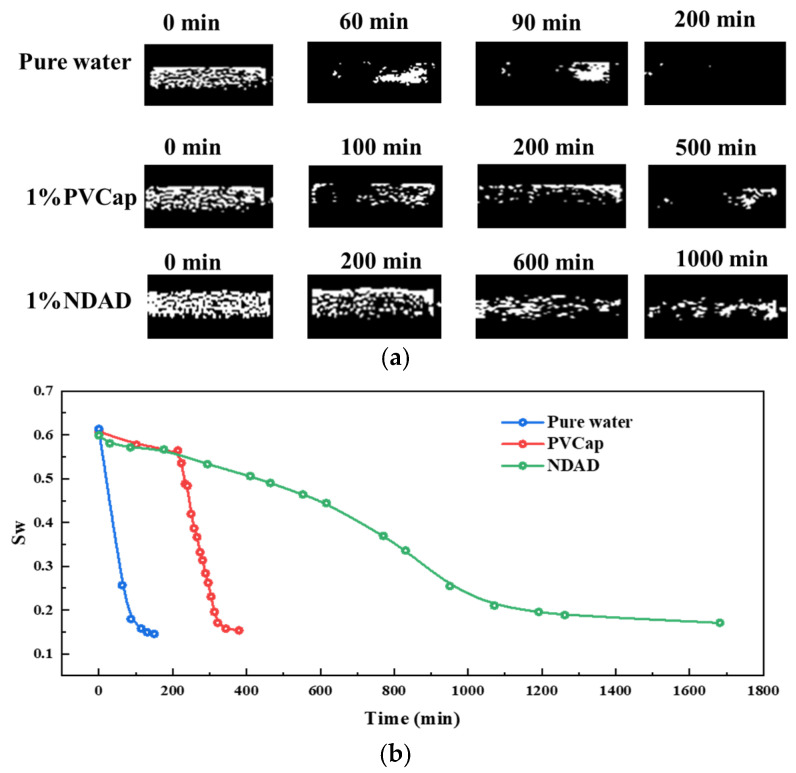
(**a**) The MRI images of hydrate formation in pure water, 1%PVCap and 1%NDAD; (**b**) The water saturation variation curve over time.

Furthermore, we conducted a visual study on the hydrate formation process using magnetic resonance imaging (MRI) technology. According to the principle of nuclear magnetic resonance, the dynamic change process of the hydrate formation process can be directly observed through the MRI results. As shown in Figure 5, the results show that in the initial stage, the fluid distribution in the porous medium is uniform, presenting as brightness throughout the entire area. With the formation of hydrates, the brightness of the nuclear magnetic resonance signal rapidly dims. This is caused by the gradual transformation of water into solid hydrates in the sample chamber, resulting in the gradual weakening of the hydrogen proton signal. In pure water, the process of hydrates formation is relatively fast. Within a very short period of time, the signal intensity within the visual filed drops rapidly, indicating the formation of hydrates. It can be found from the visualized monitoring process that the formation process of hydrates is heterogeneous. For the samples with 1%PVCap, the hydrate formation process gradually slows down. In the later stage of formation, there is still some water in the pores that does not participate in hydrate formation, which indicates that PVCap has a certain inhibitory effect on hydrate formation. For 1%NDAD, the bright area was significantly larger than that of pure water and PVCap samples within the same period of time. After the formation of hydrates was completed, there were still many signals in the middle of the samples, indicating that a considerable amount of water did not participate in the formation of hydrates, proving the good hydrate inhibition effect of NDAD.

MRI images allow us to obtain the hydrogen proton signal in the reaction chamber. According to the calibration curve, this signal value can be converted into the mass of water, which is further transformed into water saturation, Sw. According to the results of the water saturation variation curve over time, as shown in Figure 5b, it can be clearly seen that MRI quantitatively demonstrates that the water content in the reaction chamber gradually decreases during the formation of hydrates. Moreover, for different systems, the trend of water saturation decline is different.

### 2.4. Salinity Promotes Gel Transformation and Enhances Hydrate Inhibition Performance

**Figure 6:**
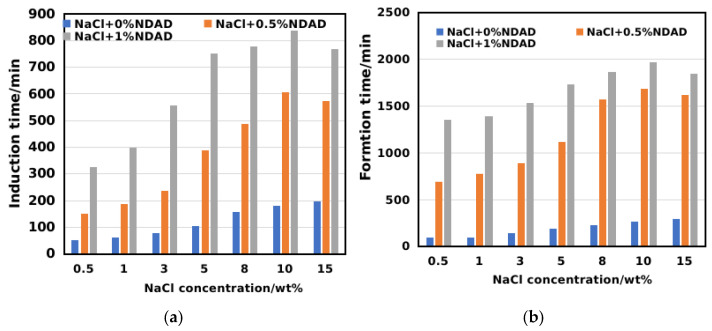
The synergistic inhibitory effect of the thermodynamic inhibitor NaCl and NDAD: (**a**) Induction time of hydrate formation; (**b**) Formation time of hydrate formation.

The synergistic inhibitory effect of the thermodynamic inhibitor NaCl and NDAD was investigated. As shown in Figure 6, the results show that for a pure NaCl solution, as the salt concentration increases, the induction time and formation time of hydrates are greater than those of pure water, which also demonstrates the inhibitory effect of thermodynamic inhibitors. However, the effect of simple thermodynamic inhibitors is not significant. After introducing NaCl into NDAD solutions of different concentrations, it can be significantly observed that with the increase in salt concentration, the induction time and formation time of hydrates show a distinct increasing trend. In a 0.5%NDAD solution, as the salt concentration increased to 10%, the induction time of the hydrate was extended to 574 min, and the formation time was extended to 1620 min. In a 1%NDAD solution, as the salt concentration increased to 10%, the induction time of the hydrate was extended to 769 min, and the hydrate formation time was extended to 1968 min. However, when the salt concentration is relatively high (15%), the inhibitory effect of the thermodynamic and kinetic combined system on hydrates decreases again.

**Figure 7:**
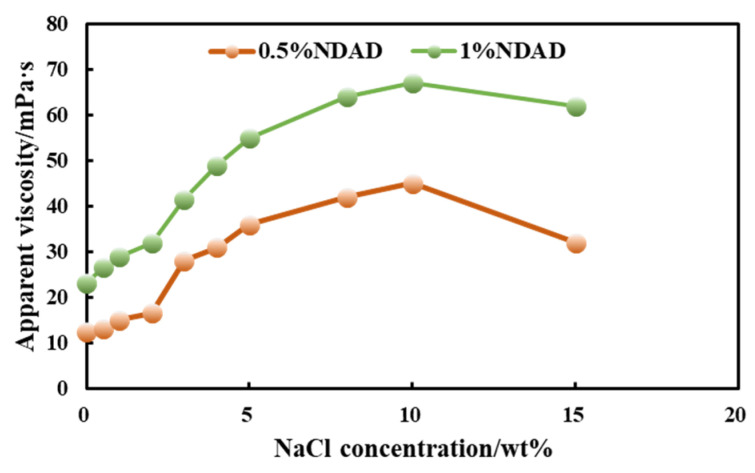
The variation curves of the apparent viscosity of NDAD aqueous solutions of different concentrations with the addition of NaCl.

Figure 7 presents the variation curves of the apparent viscosity of NDAD aqueous solutions of different concentrations with the addition of NaCl. As can be seen, as the salt concentration increases (typically within the low to medium concentration range), the polymer chains stretch, the hydrodynamic volume expands, and the friction and entanglement between molecular chains intensify, resulting in a significant increase in the solution’s viscosity. This is completely contrary to the behavior of traditional polymers (whose viscosity decreases after adding salt). This is a direct manifestation of the anti-polyelectrolyte effect in macroscopic rheological properties. Viscosity usually undergoes a process of first increasing and then decreasing as the salt concentration increases. At the optimal salt concentration (10%NaCl), the apparent viscosity values of NDAD reached their maximum values, which were 67 mPa·s at 1%NDAD and 45 mPa·s at 0.5%NDAD, respectively. At this point, the degree of chain expansion is the greatest, and the hydrodynamic volume reaches its maximum. When the salt concentration exceeds 10%, if more salt is added, the viscosity of the solution will start to decrease. This is because excessive salt ions have an overly strong shielding effect on the charges on the polymer chain, which may lead to an excessive weakening of the effective electrostatic repulsion between chain segments. It may even introduce weak hydrophobic interactions or van der Waals forces to take the lead, causing the chain to undergo a certain degree of re-contraction (but usually not to the extent of being salt-free). High ionic strength compresses the ionic atmosphere (double electric layer) around the polymer chain, reducing its effective size. A large number of salt ions interact strongly with water molecules, which may compete for the hydration layer around the polymer, reduce the solvation degree of the polymer, and indirectly affect the chain conformation and interaction.

The influence of polymer concentration on this viscosity effect was also investigated. at At higher polymer concentrations, in addition to changes in chain size, interchain interactions (such as physical entanglement, possible interchain ion pairs or hydrogen bonds) became more significant. After adding salt to disrupt intramolecular ion pairs, it may promote intermolecular interactions, further increasing viscosity or facilitating structural formation (such as gelation). However, at extremely high salt concentrations, the shielding effect will also weaken the interchain interactions.

**Figure 8:**
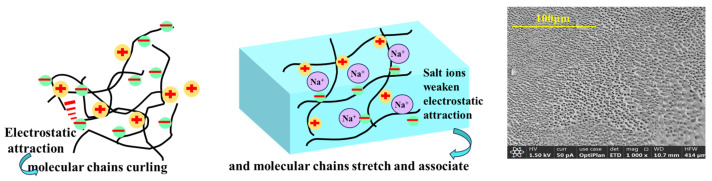
The mechanism of weak-gel structure transformation of NDAD aqueous solution by NaCl.

Figure 8 shows the mechanism of weak-gel structure transformation of NDAD aqueous solution by NaCl. In pure water, the positive and negative charged groups on the amphoteric ionic polymer chain tend to form tight intramolecular ion pairings through strong electrostatic attraction. This leads to highly contracted polymer chains, presenting a compact collapsed coil conformation. After the addition of salt, salt ions (such as Na^+^ and Cl^−^) will interact with the positive and negative charge groups associated with the polymer chain. Cations (Na^+^) competitively bind to the anionic groups (-SO_3_^−^) on the polymer chain. The anion (Cl^−^) competitively binds to the cationic groups (-N^+^(CH_3_)_3_) on the polymer chain. This competitive combination of salt ions effectively shields the strong electrostatic attraction between the positive and negative charged groups on the polymer chain, disrupting the original tight intramolecular ion pairs. As the electrostatic attraction within the molecule weakens, the originally contracted polymer chains stretch out, and the molecular size (hydrodynamic radius) increases. The chain has become more flexible and open. This conformational shift is the basis for subsequent changes in viscosity and gelation behavior.

Salt, as a thermodynamic inhibitor, has a synergistic effect with kinetic inhibitors in inhibiting the formation of hydrates. For zwitterionic polymer, the presence of salt further increases the viscosity of the polymer, transforming the coiled structure of the conventional linear polymer aqueous solution into a weak gel network structure. This further increases the mass transfer resistance of methane molecules in the solution, thereby effectively reducing the induction and formation time of hydrates.

### 2.5. Impact of Hydrate Formation Inhibition Performance of NDAD

Since the hydrate formation rate can be affected by the concentration of inhibitor NDAD, stirring rate and supercooling degree, the inhibition performance of NDAD will be affected by many factors. Therefore, a multi-factor statistical experimental evaluation method was established to explore the hydrate formation inhibition performance and influence factors of NDAD, revealing the interaction relationship between influence factors.

Response surface methodology (RSM) is a statistical method for optimizing stochastic processes. The objective is to find the quantitative rule between the target index and factors, and to obtain the optimized combination of the factors and levels. The three-dimensional surface model with multiple continuous variables can be created using RSM. Through the analysis of the surface model and corresponding contour lines, the mathematical relationship between the response value and the variable can be obtained, so as to evaluate the influence of various factors on the response value and obtain the independent or interactive relationship of each factor. Therefore, the optimal parameters can be obtained in the least number of experiments [27,28]. It has been widely used in mathematical modeling and model optimization in chemistry, physics and biology.

According to the experimental method of hydrate formation, NDAD addition (1%, 2%, 3%), stirring rate (200, 300, 400, r/min) and supercooling degree (2, 4, 6 °C) were choosen as the main influencing factors, and hydrate formation time was employed as the response value. Design Expert software was used (Version 8.0.6, Stat-Ease, Inc., Minneapolis, MN, USA) to establish a three-factor and three-level response surface experiment design through Box-Behnken design in response surface design, as shown in Table 3.

Based on the RSM statistical experimental design, 17 sets of experiments with different variable combinations were tested. The results are shown in Table 4. As the introduction of NDAD, the hydrate formation time increased rapidly, which was much higher than that in pure water, indicating that NDAD could effectively inhibit hydrate formation. In addition, the experimental conditions (stirring rate and subcooling degree) also have a great influence on the hydrate formation time.

According to the experimental results, a variety of models (linear, two-factor, quadratic equation, cubic equation) built in the software were used to fit the data. Among the fitting models, the quadratic polynomial model shows that the model is statistically significant with a lower *p*-value (<0.0001). Meanwhile, the coefficient of determination (R^2^) was close to unity, and the adjusted R^2^ values also exceeded 0.9, indicating that the regression model had a satisfactory fitting degree and prediction accuracy. Therefore, a quadratic polynomial model was selected to fit the MH formation time. The quadratic model was applied to fit the data using NDAD dosage (A, %), stirring rate (B, r/min), and supercooling degree (C, °C) as the independent variables and MH formation rate (formation time, min) as the dependent variable. The regression equation shown in the following equation could be obtained:

(1) $$\begin{array}{c} {\mathrm{Formation}\;\mathrm{time}\;}_{\mathrm{MH}}\\ =2423.75-138.05A+1.61525B-91.075C+0.035\mathrm{AB}-35\mathrm{AC}-0.3025\mathrm{BC}+368.9A^{2}-2.93B^{2}+15.24375C^{2}, \end{array}$$

Further analysis of variance and significance was conducted on the regression model, and the results are shown in Table 5. In general, a *p*-value less than 0.05 (confidence interval 95%) indicates that the model is statistically significant. A signal-to-noise ratio greater than 4 indicates that the model design precision and model response value are reasonable. The variance coefficient is the ratio of the estimated standard error to the average observed response, and a value less than 10% indicates that the model has reproducibility and high reliability. The loss-of-fit item *p*-value is greater than 0.05, and the loss of fit is not significant, indicating that the model is suitable for predicting the response within the range of study variables. As can be seen from the data in Table 5, the regression model conforms to the above test principles, all uncertainties are within the acceptable range, and the model has a good adaptability to the experimental results of hydrate formation time.

Moreover, various factors’ influence degree and interactive effect on the response value were analyzed by the F- and *p*-values. The larger the F-value, the more significant the impact of variables on the response value. As shown in Table 5, the F-values of variables A, B, and C were 300.64, 94.22, and 195.80, respectively. Thus, the influencing degree of each variable on the MH formation time was in the order of NDAD dosage (A) > supercooling degree (C) > stirring rate (B). The NDAD dosage was the main controlling factor. Additionally, the *p*-values of variables A, B, C, BC, A^2^, and C^2^ were less than 0.05, indicating that the combination of these factors significantly impacted the response value.

**Figure 9:**
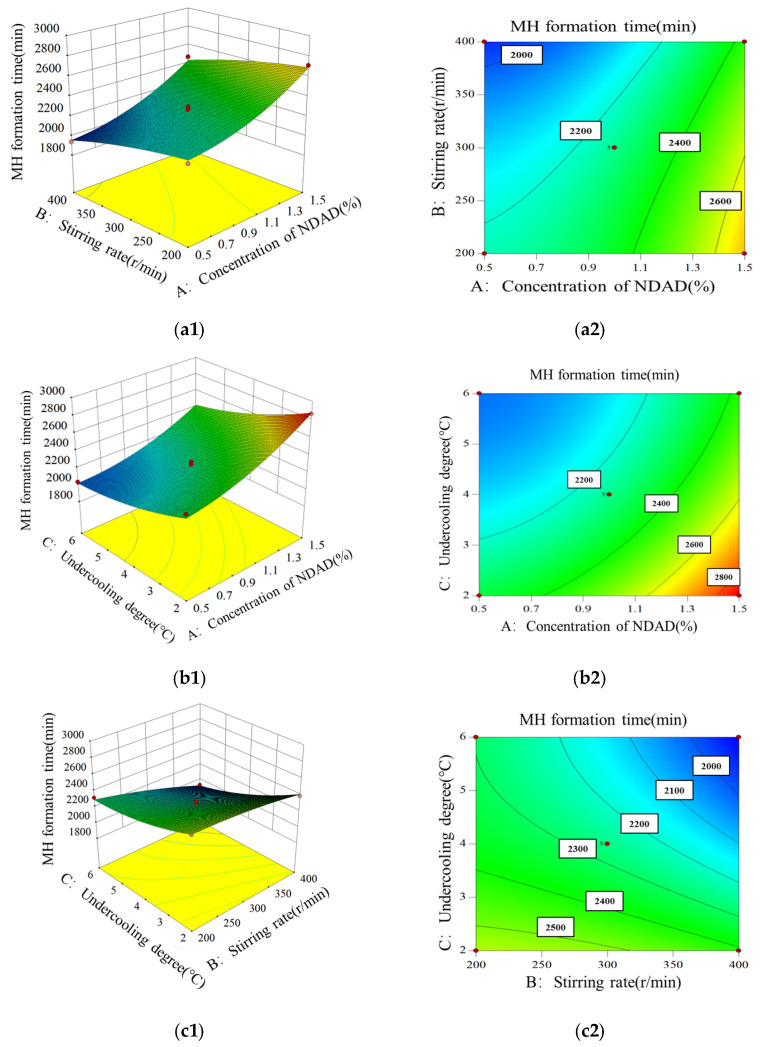
(**a1**–**c1**) 3D response surface plots of formation time of MH; (**a2**–**c2**) contour map of formation time of MH.

Additionally, combined with the regression model, the 3D response surface of hydrate formation time vs. different interactive combinations was plotted and analyzed. Figure 9(a1) shows the interactive effect of NDAD dosage and stirring rate on MH formation time. The results showed that when the stirring rate was fixed, the hydrate formation time increased rapidly with the increase in NDAD dosage, while at an unchanged NDAD dosage, the hydrate formation time showed a smooth decrease with the increase in stirring rate. The stirring rate can affect the mass transfer rate in aqueous solution. A large mass transfer rate can increase hydrate formation rate, resulting in shorter hydrate formation time. The contour plot in Figure 9(a2) gives the same conclusion. Figure 9(b1) shows the interactive effect of NDAD dosage and subcooling degree on MH formation time, and the Figure 9(b2) gives a contour plot. When the degree of supercooling was fixed, the hydrate formation rate increased rapidly with the increase in the NDAD concentration. The hydrate formation time decreased with the increase in supercooling degree. This is because the degree of supercooling is the driving force of hydrate formation. Therefore, the higher the degree of supercooling, the faster the rate of hydrate formation. Figure 9(c1) shows the interaction effect of subcooling degree and stirring rate on hydrate formation time. When the supercooling degree was fixed, the hydrate formation time decreased with the increase in stirring rate. When the stirring rate remained constant, the hydrate formation time decreased with the increase in supercooling degree. From the contour plot in Figure 9(c2), the hydrate formation time decreased with the increase in stirring rate and supercooling degree.

### 2.6. The Feasibility of NDAD in Practical Applications

Unlike PVCap’s non-degradable polyvinyl backbone, NDAD’s hydrolyzable amide bonds facilitate rapid biodegradation, aligning with offshore discharge regulations. This positions NDAD as an eco-friendly alternative for sensitive marine ecosystems.

Experimental results demonstrated that lower NDAD (about 0.2%) can achieve equivalent inhibition to 1.0 wt% PVCap, reducing chemical consumption and improving economic viability.

We evaluated the compatibility of NDAD with the drilling fluid. Herein, NDAD was added to the Na-Bent soil-based drilling fluid, and the system was named QYZ-1.

The rheological and filtration performance of QYZ-1 system was evaluated firstly. The apparent viscosity of the QYZ-1 system at room temperature is 47 mPa·s, and the filtration volume is 6.8 mL. These results indicate that the system has both excellent rheological properties and filtration performance at room temperature. Due to the low temperature conditions in marine sediments, the apparent viscosity and filtration volume under 4 °C were also evaluated. Results showed that the apparent viscosity at 4 °C is 66 mPa s, indicating that the viscosity increases at low temperature. The filtration volume shows 5.2 mL at 4 °C. The results show that NDAD has good compatibility with drilling fluid and can effectively improve the rheological and fluid loss performance of drilling fluid.

The clay hydration inhibition performance of QYZ-1 drilling fluid system was evaluated by linear expansion experiment and shale rolling recovery experiment.

**Figure 10:**
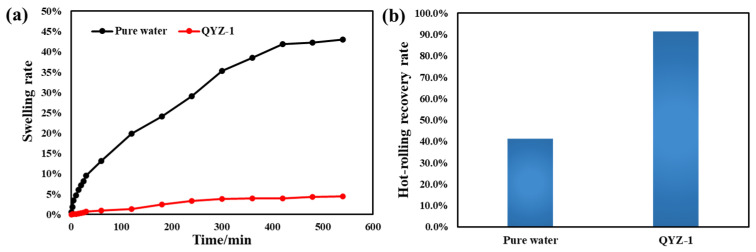
(**a**) The linear expansion experiment for pure water and QYZ-1 system; (**b**) shale rolling recovery experiment for pure water and QYZ-1 system.

Figure 10 shows the evaluation results of linear expansion of the system. The expansion rate of mud cake pressed by calcium soil in pure water is 43.98%, and the expansion rate of mud cake in QYZ-1 drilling fluid system is significantly reduced (only 4.4%). Figure 9b shows the evaluation results of hot rolling recovery rates. The recovery rate of cuttings in pure water is only 39.34%, while 90.6% can be seen in QYZ-1 drilling fluid, indicating that the QYZ-1 drilling fluid has a good ability to inhibit hydration and dispersion.

### 2.7. Hydrate Formation Inhibition Mechanism

**Figure 11:**
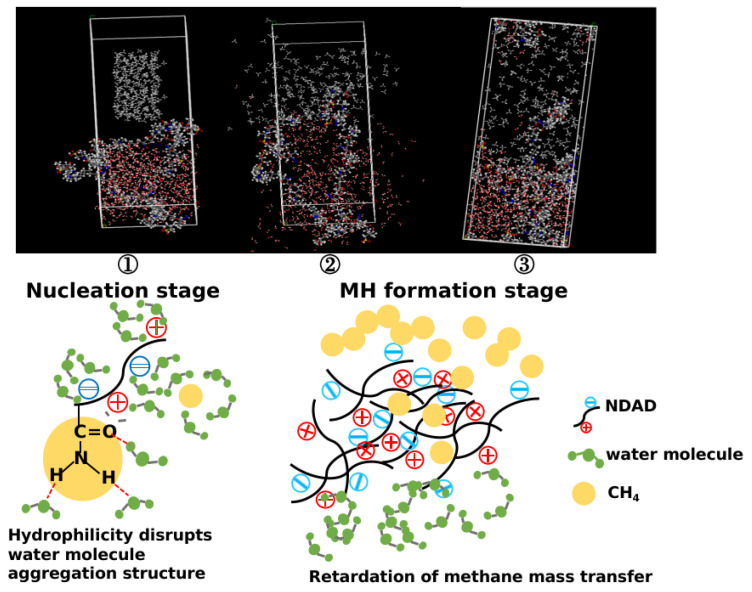
The diffusion process of methane molecules in NDAD solution.

The diffusion process of methane molecules in NDAD solution was simulated by the molecular simulation method. Figure 11 shows the initial process, assuming that the methane molecules are on the surface of the solution. After the simulation begins, the methane molecules begin to diffuse freely into the solution. From Figure 11, it can be seen that it is difficult for methane molecules to enter the solution after the simulation starts, and most methane molecules still exist above the polymer solution, which indicates that the presence of polymer increases the mass transfer resistance of methane molecules in the aqueous solution, and further leads to the difficulty of hydrate formation. As can be seen from the stage ② in Figure 11, significant numbers of water molecules adsorb around the polymer chains due to the abundance of hydrophilic and charged groups in NDAD molecules, which interact strongly with the water molecules, making the water molecules tend to move irregularly, resulting in increased difficulty in the formation of cage structures.

A significant number of water molecules adsorb around polymer chains due to abundant hydrophilic and charged groups within NDAD’s molecular structure. As established by prior analysis, NDAD mediates hydrate inhibition through dual mechanisms (Figure 9): cationic and anionic groups in NDAD exhibit intense hydration effects, leading to amide groups readily forming hydrogen bonds with water molecules, destabilizing the ordered cage structure of water and consequently inhibiting hydrate nucleation; NDAD polymer has an amphiphilic structure, and it can inhibit the mass transfer of methane molecules by increasing the solution viscosity, which can effectively slow down the embedding of methane molecules into the cage structure of water molecules and thus inhibit the hydrate formation. Furthermore, hydrate inhibition is achieved through hydrophobic interactions: methyl pendant groups on the NDAD polymer anchor into open cavities at the hydrate-aqueous interface, thereby blocking methane molecules from entering hydrate cages. The synergistic action of these dual mechanisms effectively inhibits hydrate formation [28,29,30].

## 3. Conclusions

In this paper, a zwitterionic polymer NDAD (NVCL/DMAA/AMPS/DMDAAC) was synthesized, to simultaneously inhibit hydrate formation and clay hydration.

The optimal synthesis conditions of NDAD are optimized by single factor experiments and orthogonal experiments. The optimal total monomer concentration was 19%, NVCL/DMAA/AMPS/DMDAAC = 4:2:2:2, and the initiator concentration was chosen as 0.5%, and the optimal reaction temperature was 35 °C.

NDAD exhibits an advanced performance for inhibiting the hydrate formation. Compared with the commonly used hydrate inhibitor PVCap, NDAD can extend the hydrate formation time by 4.9 times. NaCl and NDAD have a significant synergistic inhibitory effect on the formation of natural gas hydrates through forming a physical gel network. Based on the response surface method (RSM) design, a statistical significance prediction model for hydrate formation time was established, which showed that the inhibitor concentration could significantly delay the hydrate formation time and interact with the mass transfer rate (stirring rate) and the degree of supercooling. The NDAD dosage demonstrates the main control factor on hydrate formation time.

NDAD effectively hinders methane mass transfer through dual pathways: (1) adsorption onto hydrate surfaces and increased aqueous phase viscosity; (2) Simultaneously, its hydrophilic and amide groups disrupt water aggregation, thereby inhibiting methane hydrate nucleation. According to the experimental results, NDAD is an efficient hydrate inhibitor. In the future, we will be committed to conducting field tests of NDAD in deepwater drilling, further upgrading the product performance to enable it to play an effective role in solving the inhibition of deepwater hydrate formation. Meanwhile, the hybrid SiO_2_-NDAD composite material can achieve self-healing fluid networks through dynamic bonds, which is also a new idea we want to study in the future to enhance the performance of deepwater drilling fluids.

## 4. Materials and Methods

### 4.1. Materials

N-Vinylcaprolactam (NVCL, 99%), N,N-Dimethylacrylamide (DMAA,99%), 2-Acrylamido-2-methyl-1-propanesulfonic acid (AMPS, 98%), diallyldimethylammonium chloride solution (DADMAC, 60 wt% in water), ammonium persulfate [(NH_4_)_2_S_2_O_8_], and sodium hydrogen sulfite (NaHSO_3_) were purchased from Shanghai Aladdin Biochemical Technology Co., Ltd., (Shanghai, China). All reagents and solvents were commercially sourced. Calcium bentonite (Ca-Bent) was provided by Daqing Oilfield Company (Daqing, Heilongjiang, China), and sodium bentonite (Na-Bent) by Yuhong New Materials Co., Ltd., (Ningbo, Zhejiang, China). Shale cuttings were obtained from an oilfield in China. Deionized water was used for solution preparation and hydrate experiments. Methane gas (99.99% purity) for hydrate formation was supplied by Wuhan Jiuju Gas Technology Co., Ltd., (Wuhan, Hubei, China).

### 4.2. Synthesis of NDAD

**Figure 12:**
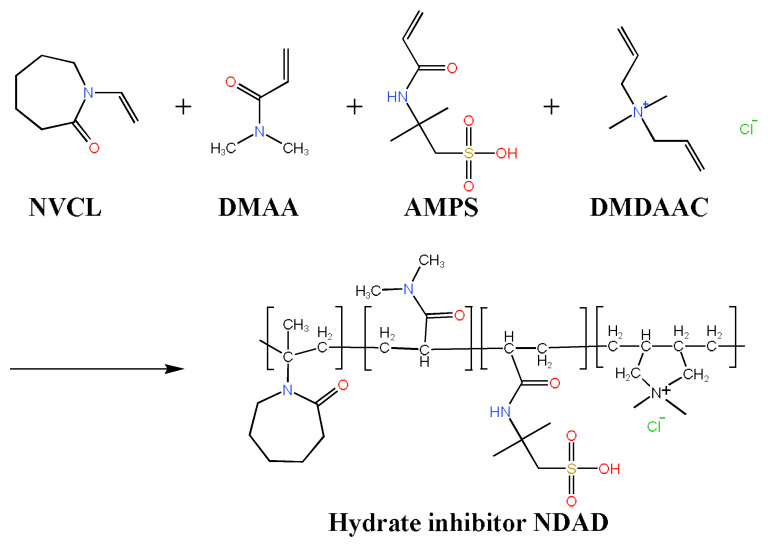
Schematic diagram of the reaction process of NDAD.

The NVCL/DMAA/AMPS/DMDAAC copolymer NDAD was synthesized via free radical polymerization using the following procedure (Figure 12). Specified amounts of NVCL, DMAA, AMPS, DMDAAC monomers and polyvinyl alcohol emulsifier were dissolved in deionized water within a three-neck flask. The mixture was stirred at 300 rpm for 15 min to ensure complete dissolution. The system was deoxygenated by nitrogen purging for 30 min, followed by heating to 35 °C. An initiator solution of (NH_4_)_2_S_2_O_8_/NaHSO_3_ in deionized water was prepared and added dropwise to the reaction vessel. Polymerization proceeded under a N_2_ atmosphere for 8 h at 35 °C. The crude product was purified by ethanol precipitation, yielding a white polymer precipitate. After vacuum drying at 50 °C, residual monomers were removed via acetone extraction, followed by final vacuum drying at 50 °C to obtain pure NDAD [5,31,32,33].

### 4.3. Structural Characterizations of NDAD

(1) Fourier infrared spectroscopy (FT-IR)

The molecular structure of NDAD was determined by FT-IR spectroscopy (KBr pellet method). Spectra were acquired over 400–4000 cm^−1^ at 4 cm^−1^ resolution.

(2) Nuclear magnetic resonance hydrogen spectroscopy (^1^H NMR)

^1^H NMR spectra were recorded at 25 °C in D_2_O using a Bruker AV 400 MHz spectrometer (Bruker, Billerica, MA, USA).

(3) Gel Permeation Chromatography (GPC)

Molecular weight parameters were determined by GPC (Agilent 1260 Infinity II LC System, Santa Clara, CA, USA) equipped with an Agilent G7110B column. Using DMF as mobile phase, 50 μL sample injections provided precise measurements of average molecular weights and molecular weight distributions.

### 4.4. Method of Hydrate Formation

High-pressure reactor method: A high-pressure constant-volume reactor was employed to assess NDAD’s hydrate inhibition performance. Details of the experimental setup can be found in our previous work [34]. The setup operates at pressures up to 30 MPa and temperatures from −20 °C to 100 °C. Temperature and pressure during hydrate formation were monitored in real time using a ±0.01 °C accuracy thermocouple and a ±0.01 MPa precision pressure transducer.

Magnetic resonance imaging(MRI): Magnetic resonance imaging technology is a non-destructive technique that can monitor hydrogen proton signals in real time. In the field of hydrate research, MRI has been widely used to detect the dynamic process of hydrate formation and decomposition. In this paper, we used MRI instruments to conduct experiments on the hydrate formation process in a sample chamber. The sample tube is processed from a special non-magnetic material and is a hollow cylindrical tube with a length of 7 cm and a diameter of 2.5 cm. Before the experiment, glass microspheres were uniformly added to it as porous media, and then the hydrate generation experiment was carried out by the excess gas method [12,35].

### 4.5. Evaluation Method for Drilling Fluid

The rheological properties and fluid loss performance of drilling fluids were evaluated in accordance with API standards.

The inhibitory properties of NDAD polymer against clay hydration were assessed through linear swelling tests and hot-rolling recovery experiments [13,14].

Linear swelling test: Calcium bentonite (10 g) was compressed under 10 MPa for 5 min to form mud cakes. Initial thickness was measured with vernier calipers. Each cake was immersed in 200 mL polymer solution within a linear swell meter, with swelling values recorded versus time. The linear expansion rate of the mud cake at any moment is calculated based on the amount of expansion data and the original thickness of the mud cake. Further, the linear expansion rate and time curve are plotted.

Hot-rolling recovery rate test: Shale drill cuttings (5–10 mesh) were used to evaluate NDAD’s ability to inhibit clay hydration and dispersion. Specifically, 30 g of cuttings and 210 mL of 1% NDAD solution were added to a hot-rolling cell. The sealed cell underwent hot-rolling at 77 °C for 16 h. After cooling, cuttings were recovered by sieving through a 40-mesh sieve, oven-dried, and weighed. The hot-rolling recovery rate was calculated as the percentage of recovered mass relative to the initial sample mass.

### 4.6. Molecular Simulation

To better investigate the hydrate formation inhibition mechanism with the effect of inhibitors [16,19,36], the molecular simulations of the mass transfer process in NDAD solution were carried out. The system used to study the mass transfer process contained water, methane, and an inhibitor molecule.

## Figures and Tables

**Table 1:** The factors and the level of orthogonal test.

Level	Factor A	Factor B	Factor C	Factor D
	Total Monomer Concentration/%	Monomer’s Molar Ratio	Temperature /°C	Initiator Concentration /%
1	15%	3:3:2:2	25	0.3
2	17%	2:2:3:3	30	0.5
3	19%	2:4:2:2	35	0.7
4	21%	4:2:2:2	40	0.9

**Table 2:** The orthogonal test results of NDAD.

Run	A	B	C	D	Yield /%	Hydrate Inhibition Rate/%	Clay Hydration Inhibition Rate/%	X	Y	Z	H
1	1	1	1	1	63.8	230%	52%	16.2%	13.7%	32.7%	0.6263
2	1	2	2	2	74.2	524%	68%	50.5%	68.5%	65.1%	1.8406
3	1	3	3	3	78.9	601%	75%	66.0%	82.8%	80.0%	2.2877
4	1	4	4	4	70.1	479%	60%	37.0%	60.1%	49.1%	1.4617
5	2	1	2	3	77.8	574%	72%	62.4%	77.7%	74.2%	2.1430
6	2	2	1	4	59.6	157%	43%	2.3%	0.0%	12.4%	0.1467
7	2	3	4	1	65.3	248%	58%	21.1%	17.1%	44.0%	0.8224
8	2	4	3	2	86.9	609%	83%	92.4%	84.4%	96.0%	2.7276
9	3	1	3	4	76.2	562%	68%	57.1%	75.5%	65.5%	1.9803
10	3	2	4	3	70.8	515%	63%	39.3%	66.8%	54.5%	1.6060
11	3	3	1	2	68.8	479%	65%	32.7%	60.2%	59.6%	1.5248
12	3	4	2	1	89.2	693%	85%	100%	100%	100%	3.0000
13	4	1	4	2	66.4	297%	50%	24.8%	26.2%	27.6%	0.7860
14	4	2	3	1	72.9	507%	62%	46.2%	65.3%	53.5%	1.6491
15	4	3	2	4	62.8	218%	62%	12.9%	11.5%	51.6%	0.7598
16	4	4	1	3	58.9	169%	37%	0.0%	2.3%	0.0%	0.0226
K_avg_	1	1.55	1.38	0.58	1.52						
	2	1.46	1.31	1.94	1.72						
	3	2.03	1.35	2.16	1.51						
	4	0.8	1.8	1.17	1.09						
optimum factor		3	4	3	2						
R		1.22	0.49	1.58	0.63						

**Table 3:** Experimental range and levels of independent variables.

Level	Factor A NDAD Concentration/%	Factor B Stirring Rate/(r/min)	Factor C Supercooling Degree/°C
−1	0.5	200	2
0	1.0	300	4
1	1.5	400	6

**Table 4:** Experimental design and results of response surface methodology.

Run	Actual Level of Variables			Response Value
	Factor A NDAD Concentration/%	Factor B Stirring Rate/(r/min)	Factor C Supercooling Degree/°C	MH Formation Time /min
1	1	400	2	2390
2	0.5	300	6	2038
3	1.5	400	4	2466
4	1	300	4	2248
5	1.5	300	2	2864
6	0.5	300	2	2378
7	1	400	6	1920
8	1	200	2	2548
9	1	300	4	2262
10	1	200	6	2320
11	0.5	200	4	2189
12	1.5	300	6	2384
13	1	300	4	2289
14	0.5	400	4	1938
15	1	300	4	2240
16	1.5	200	4	2710
17	1	300	4	2265

**Table 5:** Analysis of variance and significance analysis results of the quadratic model.

Source	Sum of Squares	df	Mean Square	F-Value	*p*-Value	Significant
Model	944,400.21	9	104,933.36	71.33	<0.0001	**
A, NDAD dosage	442,270.13	1	442,270.13	300.64	<0.0001	**
B, stirring rate	138,601.13	1	138,601.13	94.22	<0.0001	**
C, supercooling degree	288,040.50	1	288,040.50	195.80	<0.0001	**
AB	12.25	1	12.25	0.01	0.9298	
AC	4900.00	1	4900.00	3.33	0.1107	
BC	14,641.00	1	14,641.00	9.95	0.016	*
A^2^	35,812.42	1	35,812.42	24.34	0.0017	**
B^2^	3608.53	1	3608.53	2.45	0.1613	
C^2^	15,654.53	1	15,654.53	10.64	0.0138	*
Residual	10,297.55	7	1471.08			
Lack of fit	8242.75	3	2747.58	5.35	0.0695	Not significant
Pure error	2054.80	4	513.70			
Cor total	954,697.76	16				

* *p*-value < 0.05; ** *p*-value < 0.01.

## Data Availability

The original contributions presented in this study are included in the article. Further inquiries can be directed to the corresponding author.

## Abbreviations

The following abbreviations are used in this manuscript:

NDAD	Copolymer of NVCL, DMAA, AMPS, and DMDAAC
MRI	Magnetic resonance imaging
MH	Methane hydrate
RSM	Response surface methodology
NVCL	N-Vinylcaprolactam
DMAA	N,N-Dimethylacrylamide
AMPS	2-Acrylamido-2-methyl-1-propanesulfonic acid
DADMAC	diallyldimethylammo-nium chloride solution
API	American Petroleum Institute
